# Regulation of Phosphatidylethanolamine Homeostasis—The Critical Role of CTP:Phosphoethanolamine Cytidylyltransferase (Pcyt2)

**DOI:** 10.3390/ijms14022529

**Published:** 2013-01-25

**Authors:** Zvezdan Pavlovic, Marica Bakovic

**Affiliations:** Department of Human Health and Nutritional Sciences, University of Guelph, 50 Stone Road East, Guelph, Ontario, N1G 2W1, Canada; E-Mail: zpavlovi@uoguelph.ca

**Keywords:** phosphatidylethanolamine, CTP:phosphoethanolamine cytidylyltransferase, Pcyt2, lipid homeostasis, cell growth, hypertriglyceridemia, liver steatosis, obesity, insulin resistance, metabolic syndrome

## Abstract

Phosphatidylethanolamine (PE) is the most abundant lipid on the protoplasmatic leaflet of cellular membranes. It has a pivotal role in cellular processes such as membrane fusion, cell cycle regulation, autophagy, and apoptosis. CTP:phosphoethanolamine cytidylyltransferase (Pcyt2) is the main regulatory enzyme in *de novo* biosynthesis of PE from ethanolamine and diacylglycerol by the CDP-ethanolamine Kennedy pathway. The following is a summary of the current state of knowledge on Pcyt2 and how splicing and isoform specific differences could lead to variations in functional properties in this family of enzymes. Results from the most recent studies on Pcyt2 transcriptional regulation, promoter function, autophagy, and cell growth regulation are highlighted. Recent data obtained from Pcyt2 knockout mouse models is also presented, demonstrating the essentiality of this gene in embryonic development as well as the major physiological consequences of deletion of one Pcyt2 allele. Those include development of symptoms of the metabolic syndrome such as elevated lipogenesis and lipoprotein secretion, hypertriglyceridemia, liver steatosis, obesity, and insulin resistance. The objective of this review is to elucidate the nature of Pcyt2 regulation by linking its catalytic function with the regulation of lipid and energy homeostasis.

## 1. Introduction

**Figure 1:**
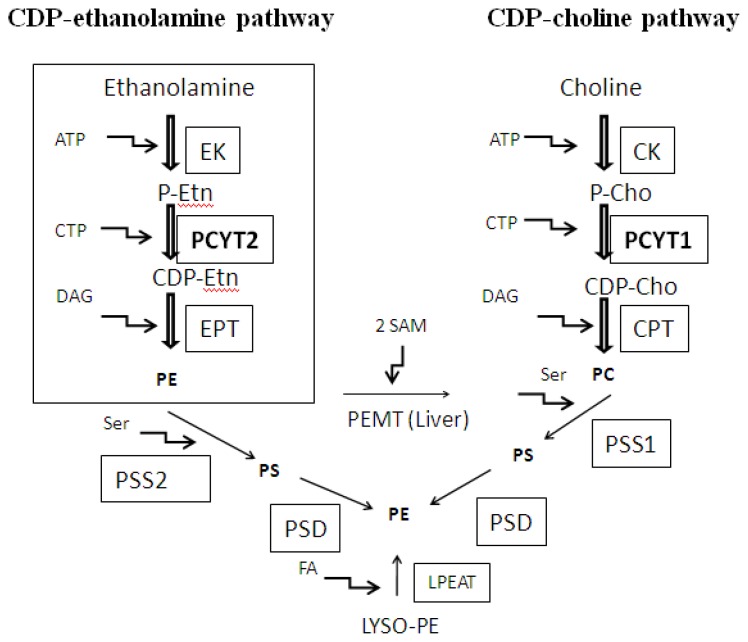
Biosynthesis of Phosphatidylethanolamine (PE). CDP ethanolamine-Kennedy pathway is the only route for *de novo* synthesis of PE. Phosphorylation of ethanolamine by ethanolamine kinase (EK) to produce phosphoethanolamine (P-Etn) is followed by the Pcyt2-mediated production of CDP-ethanolamine. The final reaction in this pathway is executed by CDP-ethanolamine:1,2-diacylglycerol ethanolaminephosphotransferase (EPT) to produce PE. The analogous enzymes of the CDP-choline brunch of the Kennedy pathway include, choline kinase (CK), CTP:choline cytidylyltransferase(Pcyt1) and CDP-choline:1,2-diacylglycerol choline phosphotransferase (CPT). In the liver PE could be transformed into PC by the action of phosphatidylethanolamine *N*-methyltransferase (PEMT). PE can also be produced in mitochondria by decarboxylation of PS by PSD. Mammals do not synthesize PS de novo; PS is produced by the head-group exchange from PE (PS synthase-2, PSS2) or PC (PS synthase-1, PSS1). In addition, PE could be made by fatty acid (FA) esterification of lyso-PE by lyso-PE acyltransferase (LPEAT).

PE is the most abundant lipid on the cytoplasmic layer of cellular membranes, with significant roles in cellular processes such as membrane fusion [1], cell cycle [2], autophagy [3], and apoptosis [4]. PE can be produced via three main biochemical pathways. Those include *de novo* production of PE via the CDP-ethanolamine Kennedy pathway, mitochondrial phosphatidylserine (PS) decarboxylation pathway (catalyzed by PS decarboxylase, PSD) and acylation of lysoPE (catalyzed by LysoPE acyltransferase, Lpeat) (Figure 1). Production and significance of PE in mammalian cells was recently reviewed [5].

The focus here is on Pcyt2, the main regulatory enzyme in the *de novo* PE synthesis via CDP-ethanolamine Kennedy pathway (Figure 1) [6]. The entering substrate in the pathway, ethanolamine, is converted into phosphoethanolamine (P-Etn) via ATP-dependent phosphorylation by the action of ethanolamine kinase (EK). Next, Pcyt2 transfers CTP to P-Etn to form CDP-ethanolamine and pyrophosphate. CDP-ethanolamine is subsequently coupled with diacylglycerol (DAG) by CDPethanolamine:1,2-diacylglycerol ethanolamine-phosphotransferase (EPT) to produce PE. The following chapters describe the most current state of knowledge on Pcyt2 regulation and function. The important roles of Pcyt2 in lipid homeostasis, cell growth and development are demonstrated through several lines of evidence obtained from *in vitro* and *in vivo* studies. Special emphasis is given to newly developed Pcyt2 knockout models and to the consequences of Pcyt2 deficiency involving dysregulation of energy homeostasis and development of lipid-related disorders.

## 2. Substrate Utilization and Activity of Pcyt2

Pcyt2 was purified for the first time from rat liver in the 1970s [7]. However, most of the studies on its catalytic properties have been performed during the last two decades. Similarly to CTP: phosphocholine cytidylyltransferase (Pcyt1), the analogous enzyme of the phosphatidylcholine (PC) branch of the Kennedy pathway (Figure 1), Pcyt2 utilizes both CTP and dCTP as a substrate [8]. Pcyt2 demonstrates high substrate specificity for P-Etn as P-Etn methyl-analogues and phosphocholine (P-chol) are weak competitive inhibitors of Pcyt2 [9,10], which demonstrates distinct functional roles of Pcyt2 and Pcyt1 [10,11]. Earlier research demonstrated that the availability of ethanolamine [9,12] and DAG [13] could limit *de novo* synthesis of PE. The availability of ethanolamine was a crucial parameter in the PE synthesis after partial hepatectomy in rat liver. Still, neither the activity of Pcyt2 nor the activities of the other enzymes of the PE Kennedy pathway were changed after partial hepatectomy [12]. Furthermore, okadaic acid, an inhibitor of protein phosphatases 1/2A, was shown to inhibit PE production via the Kennedy pathway independent of Pcyt2 [13]. Okadaic acid reduced DAG levels by 70% and under those conditions PE synthesis was limited by low DAG availability [13]. Phorbol esters such as phorbol-12-myristate-13-acetate (PMA) mimic DAG action on protein kinase C. Exposure of rat hepatocytes to PMA stimulated Pcyt2 activity, which led to increased PE synthesis [14]. Overexpression of Pcyt2 increased the level of CDP-Etn, but PE content remained unchanged since no adequate DAG was present [15]. The elevation of the intracellular DAG level after treatment with PMA and stimulation of phospholipid degradation by phospholipase C was concurrent with a decrease in CDP-Etn and coupled with an increase in PE [15]. Also, the anti-diuretic hormone, vasopressin, stimulated the incorporation of [^14^C]ethanolamine into PE in a dose-dependent manner [16]. The activity of Pcyt2 was elevated with vasopressin which together with observed high DAG levels led to an increase in PE production [16]. Altogether, those initial studies suggest a significant regulatory role of Pcyt2 in the production of PE under most physiological conditions when the amount of either ethanolamine or DAG is not limited.

Pioneer studies of Pcyt2 localization suggested that unlike Pcyt1, Pcyt2 was mainly cytosolic and not associated with cellular organelles [11]. A study on ultrastructural localization, however, revealed that Pcyt2 was not randomly distributed in liver cells [17]. Pcyt2 was concentrated in cisternae of the rough endoplasmic reticulum (RER), while nuclei, mitochondria and peroxisomes were only marginally labeled for Pcyt2 [17]. In *Plasmodium berghei*, Pcyt2 was found to be localized in the cytosol only [18]. This bimodal Pcyt2 distribution, the cytosolic and the ER bound, could be an efficient means for channeling CDP-Etn to EPT (an integral ER protein) for the terminal step of PE formation in the Kennedy pathway [19]. Similar studies however remain limited, and in light of existence of multiple Pcyt2 isoforms further research would be necessary to establish more firmly the subcellular distribution and function of this important enzyme.

## 3. Alternative Splicing of Pcyt2

**Figure 2:**
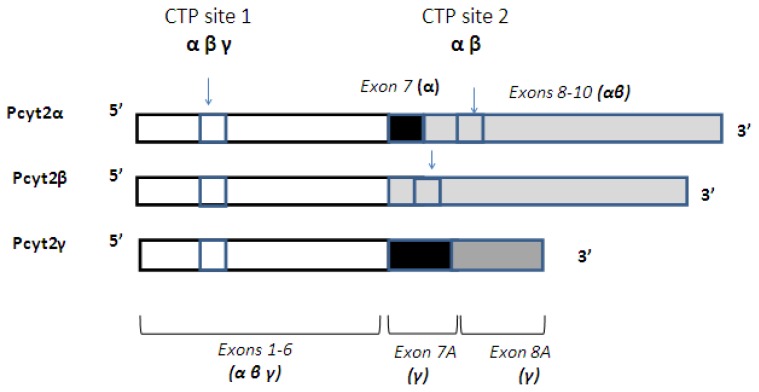
Pcyt2 mRNA splicing. Pcyt2α mRNA is composed of 14 exons. Exons 1–6 are shared with Pcyt2β and Pcyt2γ. The exon 7 is spliced from Pcyt2β due to “exon-skipping”. The rest of the mRNA from Exons 8–14 are identical in Pcyt2α and Pcyt2β. Pcyt2γ is made of 8 exons. Last two exons, depicted as Exons 7A and 8A are products of different splicing and have different structure due to retention of parts of introns 7 and 8. Region encoding the 1st putative CTP binding site is conserved in all transcripts while the 2nd putative CTP binding site is present only in Pcyt2α and Pcyt2β. Due to the different splicing mechanism and shorter transcript size, Pcyt2γ does not retain the 2nd CTP binding site.

The first Pcyt2 cDNA was cloned and characterized from yeast (*Saccharomyces cerevisiae*) from a Pcyt2-yeast mutant (ECT1) that was unable to utilize extracellular ethanolamine for PE synthesis [20]. PE accounted for less than 2% of total phospholipids in this model which confirmed an important role of *de novo* Kennedy pathway and Pcyt2 in yeast [20]. Human, rat and murine Pcyt2 were subsequently cloned [21–23]. Human Pcyt2 cDNA isolated from glioblastoma cells was able to restore the synthesis of CDP-ethanolamine as well as the formation of PE in the ECT1-deficient yeast mutant [21]. Rat Pcyt2 was shown to be 89% similar in sequence with human Pcyt2 [22]. Deducted sequences of the rat Pcyt2 protein revealed a low (~25%) similarity to other cytidylyltransferases, including human and yeast Pcyt2, rat Pcyt1 and *Bacillus subtilis* CTP:glycerol-3-phosphate cytidylyltransferase [22]. After cloning of mouse Pcyt2 gene in 2004 it was possible to establish the exon/intron structural relationship of Pcyt2 genes from various species [23]. This analysis revealed the presence of the two evolutionary conserved isoforms of Pcyt2, encoded by a single Pcyt2 gene in mouse (Figure 2). Mouse, rat, and human genes have 14 exons interrupted by 13 introns. All mammalian species retain Exon 7 in Pcyt2α while the Exon 7 is removed by alternative splicing in Pcyt2β. Both isoforms are unique cytidylyltransferases, containing two CTP binding HXGH motifs (CTP sites 1 and 2 in Figure 2) and large repetitive sequences within the *N*- and *C*-domains made by gene duplication [23,24]. Classical cytidylyltransferases such as Pcyt1 contain a single CTP catalytic site at the *N*-terminus and are not produced by gene duplication events. Although the splicing of Pcyt2 at Exon 7 was ubiquitous, the levels of the produced transcripts varied in different mouse tissues suggesting a tissue specific regulation [25]. When expressed *in vitro*, Pcyt2α had a 4-fold higher activity than Pcyt2β [25]. The isoforms differed in their catalytic properties, with Km for P-Etn 318 μM and 140 μM and the maximal velocities of 138 and 114 nmol/min/μmol for Pcytα and Pcyt2β respectively [25], showing that the availability of P-Etn could limit the function of Pcyt2β more than the function of Pcyt2α. The activity of endogenous Pcyt2 isoforms was studied in MCF7 cells [26]. The Pcyt2 activity was stimulated by serum depletion, which was demonstrated by an increase in the mRNA, protein content, and phosphorylation of Serine residues in both Pcyt2α and Pcyt2β [26].

In addition to Pcyt2α and -β variants, multiple additional Pcyt2 transcripts from rat, mouse and human are identified in the NCBI genomic databases showing the presence of a complex and species-specific regulation of Pcyt2 at the post-transcriptional level. We have recently identified a novel isoform that was expressed in all mouse tissues, named Pcyt2γ (Figure 2). Pcyt2γ resembles the isoform Pcyt2α in sharing the *N*-terminal catalytic domain and in retaining the Exon 7 sequence. Pcyt2γ, however has two alternative 3′-end splice sites in Introns 7 and 8, instead of the regular splice sites that were producing Exon 7 and 8 in Pcyt2α. Thus, a novel isoform retains parts of the Introns 7 and 8 and has a distinct exon structure and protein composition at the *C*-terminus. Different 3′ splicing sites in Pcyt2γ result in longer Pcyt2γ-specific Exon 7a and Exon 8a (Figure 2). Furthermore, Pcyt2γ does not maintain Exons 9–14 of Pcyt2α/β, while the intron retention introduces a stop codon in Exon 8a. Since the second CTP binding site is encoded by Exon 9 in Pcyt2α, this region is absent in Pcyt2γ (Figure 2). Pcyt2α and Pcyt2β mRNAs are 1881 bp and 1486 bp long and encode the proteins of 51 kDa and 49 kDa respectively. Pcyt2γ mRNA is 1228 bp long, and the predicted protein size via Expasy Swiss Institute of Biotechology Bioinformatics Resource Portal [27] is approximately 34 kDa. Future studies will demonstrate the significance of this novel variant that retains only one catalytic domain at the *N*-terminus and resembles the classical cytidylyltransferases.

## 4. Transcriptional Regulation of Pcyt2

It was shown that protein content of Pcyt2 increases 2.5-fold in rat development from day 17 of gestation to postnatal day two with the simultaneous 6-fold increase in mRNA expression, which illustrated for the first time the multiple levels of Pcyt2 regulation and its importance for embryonal development [22]. Subsequently mouse and human Pcyt2 promoters were characterized and additional types of regulation were revealed in our laboratory. Promoters of the longer mouse Pcyt2 transcript (Pcyt2α) and the shorter human Pcyt2 transcript (homologue to the mouse Pcyt2β) are localized immediately upstream of the first exon and although there is no sequence homology between the two, there is a conservation of a CAAT box at the matching distance from the transcription site as well as the consensus cis-elements for the CAAT, Sp1 and NF-Y transcription factors [23]. Both promoters are strong promoters, which is in accordance with ubiquitous expression of Pcyt2 [23].

The human Pcyt2 promoter was isolated from human breast cancer cells (MCF-7) [28]. It is TATA-less and driven by a functional CAAT box (−90/−73) and by negative (−385/−255) and positive regulatory elements (−255/−153) in the upstream regions [28]. The binding regions of the nuclear transcription factors such as NFκB, C/EBP, NF-Y and EGR1 were identified upstream of the transcription initiation site and interaction among these factors with the CAAT box was shown to regulate promoter activation and Pcyt2 transcription [28]. The activity of Pcyt2 is lower in breast cancer cells (MCF-7) in comparison to mammary epithelial cells (MCF-10A), which was confirmed at the level of promoter activity, mRNA expression, and the protein content [29]. This was found to be due to the higher protein amount and nuclear activity of the transcription factor early growth response protein 1 (EGR1) in MCF-10A cells relative to MCF-7 cells [29]. The promoter activity of Pcyt2 in MCF-7 cells was maintained by proximal CAAT and GC regions and by increased nuclear NF-κB activity. Hence, this study demonstrated the importance of EGR1 and NF-κB as transcriptional regulators of human Pcyt2 [29].

Mouse Pcyt2 was found to be transcriptionally up-regulated by serum-deficiency induced differentiation of the skeletal muscle cells C_2_C_12_ [30]. The core mouse promoter (−111/+29) was shown to be dependent on the binding of cEBP to an inverse CCAT box located at the position −82/−77 bp. Increased amount of the muscle–specific regulator, MyoD, reduced the content of Sp1 (binds to region −508/−378 bp), which together with the decrease in ratio of Sp1 to Sp3 (binds to region −157/−111 bp) was identified as responsible for the stimulation of transcription of Pcyt2 gene in differentiated C_2_C_12_ myotubes relative to undifferentiated myoblasts [30].

Liver X receptor (LXR) can also modulate promoter activity and transcription of Pcyt2 [31]. An endogenous activator of LXR (25-hydroxycholesterol (25-OH)) and the LXR synthetic agonist (TO901317) both significantly reduced the biosynthesis of PE via the CDP-ethanolamine Kennedy pathway by inhibiting the promoter function and expression of Pcyt2 in mouse embryonic fibroblasts (C3H10T1/2) and human MCF-7 cells [31]. A similar type of regulation of Pcyt2 was independently observed in NIH3T3 mouse embryonic fibroblasts cultured with various lipid agonists in the absence of serum [32]. Several serum fractions such as, low-density lipoprotein, oxysterols (25-OH, 24-OH, 27-OH-cholesterol, 24(*S*),25-epoxycholesterol) and mevalonolactate were partially responsible for the inhibition of Pcyt2 transcription, and similar to the regulation of the cholesterol biosynthetic gene, 3-hydroxy-3-methylglutaryl-CoA reductase, but not Pcyt1α [32]. Since LXR agonists modulate inflammatory responses and atherosclerosis, these studies identified Pcyt2 gene as an additional target of LXR that should be considered in future studies and LXR related drug development [31,33]. In addition, the oxysterols/LXR mediated inhibition of Pcyt2 may be an important novel type of regulation for maintaining a constant cellular ratio of PE and cholesterol [32].

## 5. The Function of Pcyt2 in Cancer Cell Growth

The biosynthesis of the membrane phospholipids including PE is of a great importance for the cell growth and progression through the cell cycle. Deregulated proliferation is a hallmark of cancer cells, thus understanding the mechanisms involved in the regulation of the major enzymes involved in phospholipid synthesis may lead to the development of novel prognostic and therapeutic strategies [33]. Pcyt2 plays a pivotal role in the production of PE plasmalogens which was reviewed in a previous publication from our laboratory [34]. Level of circulating phospholipids such as lysophosphatidylcholine (LPC) and plasmenyl-phosphoethanolamine (PE plasmalogen) were found to be depleted in ovarian cancer patients [33]. Synthetic alkylphospholipids interfere with multiple cellular processes, including phospholipid turnover and signal transduction pathways. Their primary target as cancer drugs is the plasma membrane, not the nuclear DNA which is the case with most chemotherapeutics [35]. Peroxisome proliferators (PP) modulate phospholipid levels in cancer and chronic administration of PP could increase the content of hepatic PC and PE for hepatomegaly and proliferation and cause liver cancer in rodents [36–38]. While the activity of Pcyt1 was decreased, the activity of Pcyt2 was unchanged during hepatomegaly, implying that the elevated PE was not synthesized *de novo* via the Kennedy pathway [37]. Suppression of the PE methyltransferase (PEMT) activity together with the increase in the PSD activity indicated that under the influence of PP additional liver PE was produced in mitochondria by the decarboxylation of PS (Figure 1) [37,38]. It was also reported that hexadecylphosphocholine (HePC), a lysophospholipid analogue of PC, decreased the cell growth in the liver hepatoma cells by inhibiting the activity of Pcyt1 and consequently decreasing the production of PC via the CDP-choline pathway [39]. A study by Jimenez-Lopez et al demonstrated that the synthesis of PC via CDP-choline pathway once inhibited by HePC cannot be counterbalanced by PC formed via PE methylation despite an increased PE production via CDP-ethanolamine pathway [40]. On the other hand, Pcyt2 activity could be stimulated by the anti-neoplastic drug ET-18-OCH_3_ in MCF-7 cells [41]. The increase in PE production in response to treatment with anti-neoplastic phospholipids may indicate an important role of Pcyt2 in this process. We showed that the activity of Pcyt2 was suppressed in breast cancer cells (MCF-7) cells in comparison to mammary epithelial cells MCF-10A which was evident at the level of promoter activity, mRNA expression, and protein content [29]. MCF-7 cells also increased the PE synthesis and Pcyt2 expression/activity under conditions of serum deprivation [26].

Activating transcriptional factor (ATF2) was identified as a tumor suppressor in mouse skin keratinocytes [42] and Pcyt2 was down-regulated in the ATF2 null mice, a mouse skin carcinogenesis model (Table 1). Several studies imply that Pcyt2 expression is generally reduced in different epithelium derived cancer cell lineages in comparison to normal cell counterparts [29,42,43]. Recent investigations linked diabetes mellitus and metabolic syndrome to an increased risk of colorectal cancer [44]. Pcyt2 expression was significantly reduced in invasive human metastatic colon tumor cell lines in comparison to the primary tumor cell line [43] (Table 1). Pcyt2 is up-regulated in methotrexate (MTX) resistant HT29 in comparison to MTX sensitive colon cancer cell line [45,46]. Thus, most available studies demonstrated that Pcyt2 and PE *de novo* Kennedy pathway were typically suppressed while Pcyt1 and PC *de novo* pathway was frequently up-regulated in cancers, which opens an important question on how the facilitated membrane biogenesis and bilayer PC and PE balance is achieved during facilitated cancer cell growth and tumor development. Furthermore, cancer cells seem to easily adapt to starvation conditions by up-regulating Pcyt2 activity and expression which correlated with an increased cell survival [26,46,47] and supporting the survival mechanisms could be the most critical function of Pcyt2 and PE Kennedy pathway in cancer cells.

## 6. The Role of Pcyt2 and PE in Post-Translational Modifications of Proteins

PE is the precursor of the ethanolamine phosphoglycerol moiety bound to eukaryotic elongation factor 1A (eEF1A) in *Tripanosome brucei* [59]. eEF1A plays a crucial role in binding aminoacyl-tRNAs during protein synthesis. Glu362 in the primary sequence of eEF1A is strictly conserved between the predicted amino acid sequences of mammalian, plant, and yeast cells [59]. Down-regulation of EK or Pcyt2 by RNAi decreased the amount of PE moiety in eEF1A by 30% in comparison to the control [59]. This study demonstrated that PE was the direct precursor of the ethanolamine-phosphoglycerol moiety bound of eEF1A [59]. Interestingly, conserved domains database of NCBI predicts that Pcyt2 and other cytidylyltransferases share structural similarities with the class I amino-acyl tRNA synthetases, pantothenate synthetase (PanC), ATP sulfurylase, all of which have a conserved dinucleotide-binding domain [24]. This may imply a very ancient evolutionary link between class I tRNA synthetases and the novel cytidylyltransferase superfamily. Interestingly, eEF1A was significantly overexpressed among the pancreatic cancer, leukemia and osteosarcoma cell lines, and RNA silencing against eEF1A resulted in chemosensitization toward MTX in MTX resistant HT29 cells [46]. A previous discovery of PE involvement in the elementary biological process of protein synthesis highlighted the new roles for Pcyt2 and CDP-ethanolamine Kennedy pathway [59]. Hence, the upregulation of Pcyt2 expression in MTX resistant HT29 may be important for the production of PE as a precursor of ethanolamine-phosphoglycerol moiety bound to eEF1A.

Since PE is the donor of the P-Etn moiety linking the glycosylphosphatidylinositol (GPI) anchors to proteins in procyclic form of *Trypanosomes* [60], suppression of PE synthesis may lead to impairment in GPI anchor attachment to procyclins in *Tripanosomes* [59]. In *T. brucei,* PE and PC are produced entirely through the CDP-ethanolamine Kennedy pathway [61]. Production of PE could not be compensated by decarboxylation of PS in the bloodstream form of *T. brucei*, when the CDP-ethanolamine pathway was disrupted [60]. siRNA silencing against Pcyt2 resulted in significant structural changes in the inner mitochondrial membrane topology defined by a loss of disk-like cristae, showing that the modified mitochondria was the earliest structural change observed after Pcyt2 knockdown [61]. In addition, silencing of Pcyt2 impaired the synthesis of PE and normal cell-cycle progression while oxidative phosphorylation was unaltered [61]. Therefore, by controlling *de nov*o synthesis of PE, the role of Pcyt2 extends to the regulation of mitochondrial function, protein translation and survival in *T. brucei.*

PE has an important dual role in autophagy, as a contributor to the membrane bilayers and as an autophagy-specific lipid that links microtubule-associated protein 1 light chain 3 (LC3) to the autophagosomal membrane. We demonstrated for the first time that *de novo* synthesis of all phospholipids was coupled with an increased autophagosome formation in starved liver cells, and proposed a novel role for newly formed phospholipids in the biogenesis of the initial, nascent autophagosomal membranes (known as the initiation membranes or phagophores) at the level of ER [62,63]. A modification of LC3-I (the mammalian homologue of yeast Atg8) by covalent binding of PE to form the lapidated form, LC3-II, is a crucial step for further growth and maturation of autophagosomes. We further established that ^14^C-ethanolamine incorporates into LC3-II PE and that Pcyt2 was activated during the induction of autophagy by starvation [62]. The LC3 lipidation with PE and the induction of autophagy were impaired by reduced PE synthesis in Pcyt2 deficient cells [64] which further emphasized the role of *de novo* PE pathway for the process of autophagy and cell survival under starvation conditions.

Interestingly, unlike most phospholipids, PE has been recently identified as a single endogenous nuclease-resistant cofactor in brain capable of inducing the propagation of prions in the absence of nucleic acids in hamster, sheep, mouse and vole (*Muridae*) [65]. PE alone facilitated conversion of purified recombinant mammalian prion protein (recPrP) substrate into infectious recPrP(Sc) molecules. Interestingly, a synthetic PE-plasmalogen demonstrated a strong ability to cause prion propagation. The nature of this interaction remains unknown and arouses a question of the role of PE and PE-plasmalogens in protein misfolding and pathologies of the central nervous system.

## 7. The Essentiality and Anti Obesity Function of Pcyt2

Several lines of evidence demonstrated the importance of Pcyt2 in growth and development. Pcyt2 is expressed together with genes important for human embryonic stem-cell self-renewal and differentiation [66]. Pcyt2 expression is up-regulated in pre-implantation mouse embryos in cell culture [67] and activated during muscle cell differentiation [30] Pcyt2 was also down-regulated by inhibition of histone deacetylase-HDAC and microRNA transcriptional repression during oligodendrocyte differentiation [68]. A null mutation in Pcyt2 caused embryo abortion before the octant stage in *Arabidopsis thaliana* which demonstrates the essentiality of this gene in plants [69]. Complete knockout of Pcyt2 in mice (*Pcyt2^−/−^*) caused embryonic lethality before day 8.5 which led to the conclusion that Pcyt2 is essential for mammalian development [70].

*Drosophila* (fruit fly) does not synthesize cholesterol and PE, as the most abundant phospholipid, is the regulator of fatty acid synthesis and sterol regulatory element binding protein (SREBP) activation by protease cleavage and processing [71]. Thus, PE is able to completely substitute the role of cholesterol which executes the same regulatory function in mammalian cells [71]. The protease activation of SREBP in response to treatment with palmitate is inhibited in a feedback fashion by PE [71]. Palmitate must be converted into PE to inhibit SREBP cleavage in *Drosophila* [71]. Conversion of palmitate occurs through ceramide-sphingolipid pathway. In the final step of this pathway, sphingosine-1-phosphate (S1P) became broken by S1P lyase into P-Etn and *trans*-2-hexadecenal. P-Etn is then converted by Pcyt2 to CDP-Etn and utilized for PE synthesis by the Kennedy pathway [71]. It has been recently established that *Drosophila easily shocked (eas)* mutants experience tachycardia and defects in cardiac relaxation due to perturbations in PE *de novo* synthesis and SREBP function [72]. *eas* encodes EK, the first enzyme in the CDP-ethanolamine pathway and silencing of *pect* (Pcyt2) or *cept* (choline-ethanolamine phosphotransferase) also led to *eas* null *(eas^2^)*-like heart phenotype [72]. This was accompanied with an increase in triacylglycerol (TAG) formation in heart as well as in the whole tissue sample of *eas^2^* mutants. The expression of the major genes involved in *de novo* fatty acid synthesis by lipogenesis, acetyl-coA carboxylase (*ACC*), ATP citrate lyase (*ATPCL*), and fatty acid synthase (*FAS*), were up-regulated after disturbance of PE homeostasis which led to the observed increase in TAG level. RNAi knockdown of *ACC*, *ATPCL* and *FAS* normalized TAG level which confirmed that perturbations of PE homeostasis in the fruit fly are executed through the elevation of SREBP regulation of lipogenesis [72]. The up-regulated lipogenesis and SREBP expression were first established in the heterozygous *Pcyt2^+/−^* mouse [70] and confirmed in the conditional liver-specific *Pcyt2^−/−^* knockout mice [73].

A systemic deletion of Pcyt2 and the production of complete *Pcyt2^−/−^* knockout mice were found to be embryonically lethal, confirming the essentiality of this gene for embryonic development [70]. Although *Pcyt2^+/−^* heterozygous mice are viable, they experience numerous metabolic defects as adults and during ageing [74]. The impairment of mitochondrial PE production after disruption in Psd gene also causes embryonic lethality [75]. The alternative supply of PE via the CDP-ethanolamine pathway could not substitute for the complete lack of Psd however *Psd^+/−^* mice were normal due to a compensatory increase in Pcyt2 activity and PE formation by the Kennedy pathway [75]. On the other hand, the compensatory production of PE via Psd pathway was absent in the *Pcyt2^+/−^* mice [70]. Despite the anticipated 50% decrease in Pcyt2 expression in *Pcyt2^+/−^* mice, transcriptional up-regulation of the remaining functional allele was detected. Regardless of this up-regulation of Pcyt2, the flux through the PE Kennedy pathway was reduced in the *Pcyt2^+/−^* mice. The total PE levels however were unaltered because the PE degradation was also reduced [74].

In the liver-specific *Pcyt2^−/−^* knockout PE content was only 50% reduced since mitochondrial PS decarboxylation partially compensated for the complete lack of Pcyt2 [73]. The phenotype of the liver *Pcyt2^−/−^* knockout mice showed no signs of liver injury but these animals experienced massive accumulation of liver triglycerides (TAG) [73]. On the other hand, although the phenotype of *Pcyt2^+/−^* mice is identical with the littermate controls during the first 2 months of age, Pcyt2 ^+/−^ mice progressively gaine weight which at 24–28 weeks of age leads to the development of adult-onset hypertrigyceridemia, liver steatosis and obesity [74]. Although there was no differences in food consumption between Pcyt2 heterozygots and wild type littermate mice, energy consumption is decreased in young and adult *Pcyt2^+/−^* mice [74] showing a very early defect in energy metabolism. Younger *Pcyt2*^+/−^ mice experience elevated expression of SREBP1 and lipogenesis; however, they develop fatty liver, obesity, and insulin resistance at later stages [74]. The liver-specific Pcyt2^−/−^ knockout develops steatosis but neither obesity nor insulin resistance, showing that multiple-organ Pcyt2 deficiency was a critical factor for development of the metabolic disease phenotype.

As a consequence of the reduced PE synthesis in *Pcyt2^+/−^* mice, the availability of DAG increases which results in increased production of TAG [74]. Interestingly, while only the liver PE was found to have a higher saturated fatty acid content, total polyunsaturated fatty acids (PUFA) content in PE and PS, but not in PC, decreased [70]. Fatty acid profile analysis in Pcyt2^−/−^ liver specific knockout revealed high content of stearic acid (18:0) paired with a PUFA and low presence of PE containing palmitate (16:0) paired with a PUFA [73]. Changes in the phospholipid side chains influence membrane fluidity and cell signaling which might contribute to the development of Pcyt2 deficiency phenotypes. Radiolabeling experiments *in vivo* and in primary hepatocytes culture isolated from *Pcyt2*^+/−^ mice uncovered elevated formation of DAG and TAG [76]. Increased turnover of DAG was consistent with *de novo* fatty acid synthesis and subsequent accumulation of TAG. Overexpression of Pcyt2 in primary hepatocytes resulted in increased Pcyt2 protein expression, reduced fatty acids, DAG and TAG synthesis and normalized PE synthesis and turnover [76].

Mechanisms behind hypertriglyceridemia in *Pcyt2*^+/−^ mice were recently investigated [77]. *Pcyt2^+/−^* mice (8 week-old) had normal plasma glucose, insulin, and lipoprotein content [74]. The increase in very low-density lipoproteins (VLDL) secretion corresponded with a 3-fold increase in the activity of microsomal triglyceride transfer protein (MTP) activity in 42-week old mice [77]. MTP has a crucial role in the assembly of apoB-lipoproteins [78]. In addition, a two-fold increase in chylomicron (CMs) fractions was found in enterocytes isolated from *Pcyt2^+/−^* mice which was consistent with the increase in plasma lipoproteins secreted *in vivo*. Furthermore, plasma TAG clearance of both VLDL and CMs was significantly delayed as a consequence of reduced hepatic lipase and lipoprotein lipase (LPL) expression and activities coupled with the increase in a plasma content of a potent LPL inhibitor, Angptl4. Elevated postprandial TAG level was additionally found to be a consequence of the increased expression of genes involved in intestinal lipid absorption, transport and chylomicron secretion such as fatty acid transport (*CD36*) and esterification (*FATP4*) and chylomicron formation (*MTP*). These data indicate that hypertriglyceridemia resulting from a single Pcyt2 allele knockout is a consequence of elevated lipid absorption by the intestine, increased VLDL secretion from the liver, reduced plasma TAG degradation and impaired TAG utilization by peripheral tissues [77]. All available data from Pcyt2 deficient mice demonstrate robust metabolic changes in these animals which confirmed a critical role of this gene in TAG metabolism and energy homeostasis.

## 8. Pcyt2 Expression in the Metabolic Syndrome and Related Disorders

Multiple expression data available through GEO Database of NCBI provide the evidence for regulatory role of Pcyt2 in lipid and energy metabolism disturbance of which could lead to metabolic syndrome and related metabolic disorders. All the data discussed herein are summarized in Table 1. In C_2_C_12_ cells (mouse myoblasts), palmitate treatment leads to decreased expression of PPAR coactivator 1 (PGC-1) [47]. Microarray data show that Pcyt2 is up-regulated after the treatment of C_2_C_12_ cells with palmitate. Palmitate induces inactivation of AMPK which leads to defective autophagy and generation of mitochondrial reactive oxygen species (ROS) in hematopoietic cells [79]. During autophagy, LC3-I, an important mammalian autophagosomal protein, becomes covalently bound to PE on the pre-autophagosomal membrane and remains bound through the maturation process of the autophagosome [80]. Nanoparticles such as Fullerol (C60(OH)24) cause the accumulation of polyubiquitinated proteins and facilitate autophagic cell death without triggering apoptosis in umbilical vein endothelial cells [48]. Microarray data show that Pcyt2 is up-regulated in these cells after the treatment with Fullerol. We previously showed that PE synthesis is coupled with autophagosome formation [62]. Activation of AMPK is a well-known trigger of autophagy and it was recently shown that mitochondrial ROS might actually regulate starvation triggered AMPK activation [81]. On the other hand, the AMPK activator AICAR was shown to activate AMPK while simultaneously decreasing the activity of Pcyt2 in a dose dependent manner [82]. Since PGC-1 was found to be required for AICAR-induced expression of mitochondrial proteins and GLUT4 protein in mouse skeletal muscle, it was suggested that PGC-1 mediates AMPK induced regulation of these proteins [83]. The activity of Pcyt2 has been proposed to be regulated by post-translational mechanisms mediated via AMPK under the condition of AMPK stimulation by AICAR [82]. Being a precursor of ceramide, palmitate is a potent trigger for insulin resistance in skeletal muscle. AICAR treatment was recently shown to inhibit ceramide biosynthesis and improve insulin resistance in skeletal muscle [84]. Hence, the observed up-regulation of Pcyt2 expression level that is accompanied by decrease in PGC1α expression in C_2_C_12_ treated with palmitate may imply a role of PGC-1a in transcriptional suppression of Pcyt2 and/or it may represent a compensatory increase in the expression of Pcyt2, as a response to the impaired Pcyt2 activity by the palmitate induced inactivation of AMPK. In addition, Pcyt2 is a target gene of histone deacethylase-HDAC activity and, as mentioned before, it becomes down-regulated by HDAC and microRNA transcriptional repression during oligodendrocyte differentiation [68]. Proteins from the class III family of histione deacetylases (HDACs), also known as sirtuins, are important regulators of energy homeostasis. SIRT1 is a NAD^+^-dependent protein deacetylase that belongs to this family of enzymes [50]. Pcyt2 expression is up-regulated in the liver specific SIRT1 null mice [50]. SIRT1 interacts with PPARα and it is required for the activation of PGC-1α which once again may imply a link between PGC-1α and transcriptional regulation of Pcyt2. On the other hand, when challenged with a high-fat diet (HFD), liver-specific SIRT1 knockout mice develop hepatic steatosis, hepatic inflammation, and endoplasmic reticulum stress regardless of the up-regulation of Pcyt2.

Pcyt2 expression was found to be decreased in insulin resistant muscle of obese, non-diabetic Prima Indians in comparison to its expression in insulin sensitive muscle of equally obese Prima Indians [49]. As mentioned before, Pcyt2 deficiency in *Pcyt2*^+/−^ mice have impaired tolerance to glucose and insulin at later stages of development [74]. Although the role of Pcyt2 in the development of insulin resistance in muscle is evident, elucidation of molecular mechanisms behind it require further studies that should take into account the numerous factors involved in multiple levels of regulation.

Differential gene expression profile between interscapular brown fat tissue and epididymal white fat tissue was studied in C57Bl/6 mice to get an insight into brown fat cells differentiation [51]. Brown fat cells are specialized to dissipate energy and can counteract obesity; however, it is unknown which transcriptional regulators influence adipose tissue to obtain this phenotype. Pcyt2 was up-regulated in brown adipose tissue as compare to the white fat tissue in this study. Another study showed that 3–4 months old male Bl6 mice experience remodeling of white adipose tissue toward the tissue with expended catalytic activity when treated with an agonist of β-(3)-adrenergic receptors(CL 316243) up to 6 days [52]. The number of mitochondria increased over the course of 6 days which was correlated with the up-regulation of genes involved in fatty acid oxidation and mitochondrial electron transport activity. On the other hand, Pcyt2 expression level decreased from day 0 to day 6. It is known that cold exposure and β-3-adrenergic receptor signaling robustly induce PGC-1α expression [85], which may additionally demonstrate the negative correlation between the expression pattern of PGC-1α and Pcyt2.

Several studies show that deregulation of Pcyt2 expression seems to result in hazardous consequences on metabolic parameters and liver health. A study of the remodeling of adipose tissue in mice reported that genes involved in vascularization and tissue remodeling control susceptibility to obesity [53]. Inguinal adipose tissue of mice fed high fat diet (HFD) was analyzed and Pcyt2 was found to be up-regulated in high fat gainers on HFD in comparison to low fat gainers on the same diet. Pcyt2 expression was also up-regulated in proximal and middle part of small intestine in mice fed high fat diet (HFD) for 2–8 weeks in comparison to the control animals fed low-fat diet [54]. Immunochemical analysis revealed that villi in the small intestine were enlarged in mice fed HFD. In these mice, the number of cells per villus and body length was higher, a characteristics that may function to extend the capacity of lipid absorption [54]. Increased cell proliferation requires increased phospholipid synthesis which could explain up-regulation of Pcyt2 expression. When fed a HFD for 16 weeks, Wistar rats tend to develop either obesity prone (OP) or obesity resistant (OR) phenotypes [55]. The HFD caused no difference between OP and OR rats in plasma TAG content; however, the accumulation of TAG in liver of OP rats was significantly higher than that in OR rats and the control group [55]. Pcyt2 was up-regulated in OR rats in comparison to OP rats showing that the expression level of Pcyt2 negatively correlated with the fat deposition in the liver. Disturbance in PC and consequently in PE homeostasis through the deletion of *Pemt* was previously linked to liver failure in mice [86] (Figure 1). Fat deposition in the liver was shown to be a direct consequence of the reduced Pcyt2 activity in *Pcyt2^+/−^* heterozygot mice [74] or abolished Pcyt2 function in Pcyt2 liver specific knockout mice [73]. Pcyt2 is down-regulated in liver of sphingosine-1-phosphate (S1P) lyase null mice [56]. The cleavage of sphingoid base phosphates by S1P lyase to produce P-Etn and a fatty aldehyde is the final degradative step in the sphingolipid metabolic pathway. Interestingly, even though TAG level in serum and TAG storage in the liver were elevated, adiposity was reduced in the S1P lyase deficient mice [56]. Of the major plasma membrane glycerophospholipids, PE levels were the most substantially changed, showing 30% decrease in the liver of S1P null mice [56]. Thus, decreased expression of Pcyt2 likely contributed to the observed decrease in PE level in S1P null mice. Another study showed that Pcyt2 mRNA was reduced in a mouse model of Wilsone disease, a severe metabolic disorder characterized by significant liver damage caused by genetic inactivation of copper-transporter, ATP7B [57].

Thiamine is an essential cofactor in carbohydrate metabolism and individuals suffering from diabetes and/or metabolic syndrome are generally thiamine deficient [87]. Thiamine treatment in duration of 51 weeks was shown to prevent polyphagia-induced obesity in OLETF, type II diabetic rats. Thiamine mitigated visceral adipocyte hypertrophy, liver steatosis, and skeletal muscle insulin resistance without causing damage to heart or kidneys and microarray data show that Pcyt2 was up-regulated in liver of the rats who received thiamine treatment [58]. In was shown in the early 1970’s that the administration of thiamin leads to an increase in the synthesis rate for the phospholipids with no difference between the synthesis rates for PC and PE [88]. Early life-stage mortality in lake trout was found to be caused by inadequate levels of key fatty acids in TAG and PLs in eggs, along with variable thiamine content [89]. The up-regulation in Pcyt2 expression may contribute to the *de novo* production of PE which may play a role in the observed protective effects of thiamine including the decrease in liver fat deposition.

## 9. Conclusions

As demonstrated via several lines of evidence obtained from cell culture and animal models, Pcyt2 plays a pivotal role in the execution of the processes deregulation of which could lead to the development of obesity, insulin resistance, liver steatosis and dyslipidemia. Mechanisms behind the balance between cell growth and phospholipid homeostasis are largely unexplored. Pcyt2 is a gene that is involved in the regulation of cell growth and metabolic homeostasis, and may play an important role at the intersection of these processes. Clarification of the molecular mechanisms that regulate the balance among cancer cell survival, invasiveness, and energy metabolism, may lead to the discovery of novel prognostic tools and anti-cancer strategies. As a gene that is critical for cell growth and regulation of lipid homeostasis and has been shown to be responsive to changes in the nutritional environment, Pcyt2 should be considered when developing novel approaches in the treatment of metabolic disorders and cancer.

## Figures and Tables

**Table 1:** Pcyt2 expression in lipid-related disorders and cancer.

GEO Reference	Pcyt2 Expression	Study summary	Reference
GDS2648/1420493_a_at/ Pcyt2/Mus musculus /The effect of palmitate on myoblast cell line	Up-regulated in the presence of palmitate	Analysis: C2C12 myotubes Function: Palmitate decreases the expression of PPAR co-activator 1 (PGC-1) which increases lipogenesis. Research goal: To determine a link between over-nutrition, obesity, and PGC-1 expression	[47]
GDS1553/209577_at/ PCYT2/Homo sapiens/Fullerene effect on vascular endothelial cells	Up-regulated in cells treated with fullerane	Analysis: Umbilical vein endothelial cells Function: Nanomaterials are known for its ability to potentially injure endothelial cells which may cause cell death. Research goal: To get an insight into the effects of a nanomaterial (fullerenes) on endothelial injury and toxicity.	[48]
GDS157/D84307_at/ PCYT2 /Homo sapiens/Type 2 diabetes and insulin resistance (HuGeneFL)	Down-regulated in insulin-resistant (IR) muscle	Analysis: Vastus lateralis muscle samples of insulin-sensitive and IR equally obese, non-diabetic Pima Indians. Research goal: Identification of differentially expressed skeletal muscle genes in insulin resistance.	[49]
GDS3666/A_51_P432504 /Pcyt2/Mus musculus/SIRT1 deficiency effect on the liver	Up-regulated in Sirtuin (SIRT1) null mice	Analysis: Liver specific SIRT1 knockout (SIRT1 LKO) C57BL/6 mice fed ad libitum Study goal: To examine the role of SIRT1 in the regulation of hepatic lipid homeostasis. Function: SIRT1, a NAD+-dependent protein deacetylase, is a significant regulator of energy metabolism in response to changes in the availability of nutrients.	[50]
GDS2813/1420493_a_at/ Pcyt2/Mus musculus/Brown adipose tissue	Up-regulated in brown adipose tissue in comparison to white adipose tissue	Analysis: Interscapular brown fat tissue and epididymal white fat from male C57Bl6 mice. Research goal: To identify differential gene expression profiles between brown and white adipose tissue.	[51]
GDS1225/103914_at/ Pcyt2/Mus musculus/White adipose tissue remodeling: response to β3-adrenergic receptor activation	Expression level decreases from day 1 to day 6 of treatments	Analysis: Epididymal white adipose tissue from 3 to 4 months old male Bl6 mice treated with an agonist of beta(3)-adrenergic receptors(CL 316243) for 0, 1, 3 or 6 days. Function: CL 316243 caused remodeling of white adipose tissue and expanded its catabolic activity. Research goal: Investigation of potential anti-diabetic and anti-obesity effect of CL 316243.	[52]
GDS1225/103914_at/ Pcyt2/Mus musculus/White adipose tissue remodeling: response to β3-adrenergic receptor activation	Expression level decreases from day 1 to day 6 of treatments	Analysis: Epididymal white adipose tissue from 3 to 4 months old male Bl6 mice treated with an agonist of beta(3)-adrenergic receptors(CL 316243) for 0, 1, 3 or 6 days. Function: CL 316243 caused remodeling of white adipose tissue and expanded its catabolic activity. Research goal: Investigation of potential anti-diabetic and anti-obesity effect of CL 316243.	[52]
GDS2319/357445/Pcyt2/ Mus musculus/High and low weight gainers: adipose tissue	Up-regulated in adipose tissue of high-weight gainers	Analysis: Inguinal adipose tissue of C57BL/6J males exhibiting high or low weight gain after 4 weeks on a high-fat diet. Function: Genes involved in vascularization and tissue remodeling control susceptibility to obesogenic phenotype. Research goal: To examine the role of epigenetic mechanisms in the susceptibility to obesity.	[53]
GDS3357/1420493_a_at/ Pcyt2/Mus musculus/High dietary fat effect on small intestine: time course	Up-regulated in distal, proximal and middle part of small intestine in animals fed high fat diet	Analysis: Small intestines of male C57BL/6J rodents fed a powdered high-fat purified diet for up to 8 weeks. Research goal: To examine the array of genes involved into molecular mechanism of diet induced obesity and insulin resistance and the role of small intestine in these processes.	[54]
GDS3677/rn7664/Pcyt2/ Rattus norvegicus/Highfat-diet model: liver	Up-regulated in obesity-resistant rats	Analysis: Hepatic transcript profile using cDNA microarrays in Obesity-prone(OP) and Obesity resistant(OR) phenotypes in Wistar rats on HFD for 16 weeks. Research goal: mRNA and metabolomic profiling of OP *vs* OR.	[55]
GDS3654/1420493_a_at/ Pcyt2/Mus musculus Sphingosine 1-phosphate lyase deficiency effect on liver	Down-regulated in S1P null mice	Analysis: Liver of mice (C57BL6/129sv) lacking sphingosine 1-phosphate lyase (S1P). Littermate *Sgpl1*^+/+^(wild-type) and *Sgpl1*^+/−^ mice were used as controls. Function: S1P lyase controls the final step in shingolipid degradation to produce P-Etn and a fatty aldehyde. Research goal: To establish the link between the level of shingolipids and metabolic diseases.	[56]
GDS2509 1420493_a_at/Pcyt2/ Mus musculus/Wilsone disease model	Down-regulated in ATP7B null mice	Analysis: Livers of copper-transporting ATPase ATP7B null animals. Function: Genetic inactivation of ATP7B causes Wilson’s Disease (WD), a severe metabolic disorder associated with intracellular copper overload. Research goal: To provide insight into the initial events of copper-dependent liver pathology in WD.	[57]
GDS3682/256066/Pcyt2/ Rattus norvegicus/Thiamine effect on liver in type-2 diabetic rats (OLETF rats)	Up-regulated by Thiamine supplementation	Analysis: Liver blood parameters and cardiac functions were monitored in OLETF male rats on thiamine treatment for 51 weeks. Function: Thiamine treatment influenced obesity through the reduction of visceral adipose tissue. Research goal: The impact of thiamine supplementation on obesity and metabolic disorders in rats.	[58]
GDS3330/209577_at/ PCYT2/Homo Sapiens/Methotrexate resistance in cancer	Up-regulated in resistant in comparison to sensitive HT29 cells	Analysis: Cancer cells sensitive or resistant to methotrexate (MTX). Function: MTX is used in the treatment of cancer, but long term treatment may lead to drug resistance. Research goal: Networking of the genes differentially expressed in cell lines resistant to MTX.	[45,46]
GDS3334/2630092/Pcyt2/ Mus musculus/Skin carcinogenesis model	Down regulated in ATF2 null in comparison to WT	Analysis of papillomas initiated by DMBA/TPA treatment of epidermal keratinocytes deficient for activating transcriptional factor 2 (ATF2). Function: ATF2 regulates transcription in response to stress and growth factor stimuli Resarch goal: To get an insight into the role of ATF2 in skin cancer.	[42]
GDS756/209577_at/ PCYT2/Homo sapiens	Down-regulated in metastatic colon tumor	Analysis: Differential gene expression between SW480, a primary tumor colon cancer cell line, and SW620, an isogenic metastatic colon cancer cell line. Research goal: To get insight into the progression of cancer from primary tumor growth to metastasis.	[43]
